# Preconditioning-Like Properties of Short-Term Hypothermia in Isolated Perfused Rat Liver (IPRL) System

**DOI:** 10.3390/ijms19041023

**Published:** 2018-03-29

**Authors:** Norma Alva, Raquel G. Bardallo, David Basanta, Jesús Palomeque, Teresa Carbonell

**Affiliations:** Department of Cell Biology, Physiology and Immunology, Universitat de Barcelona, Avda Diagonal, 643, 08028 Barcelona, Spain; nvalva@ub.edu (N.A.); rgomezbardallo@ub.edu (R.G.B.); basanta_89@yahoo.es (D.B.); jpalomeque@ub.edu (J.P.)

**Keywords:** glutathione, hypothermic preconditioning, ischemia/reperfusion injury, lipid peroxidation

## Abstract

Hypothermia may attenuate the progression of ischemia-induced damage in liver. Here, we determined the effects of a brief cycle of hypothermic preconditioning applied before an ischemic/reperfusion (I/R) episode in isolated perfused rat liver (IPRL) on tissue damage and oxidative stress. Rats (male, 200–250 g) were anaesthetised with sodium pentobarbital (60 mg·kg^−1^ i.p) and underwent laparatomy. The liver was removed and perfused in a temperature-regulated non-recirculating system. Livers were randomly divided into two groups (*n* = 6 each group). In the hypothermia-preconditioned group, livers were perfused with hypothermic buffer (cycle of 10 min at 22 °C plus 10 min at 37 °C) and the other group was perfused at 37 °C. Both groups were then submitted to 40 min of warm ischemia and 20 min of warm reperfusion. The level of tissue-damage indicators (alanine amino transferase, ALT; lactate dehydrogenase, LDH; and proteins), oxidative stress markers (thiobarbituric acid-reactive substances, TBARS; advanced oxidation protein products, AOPP; and glutathione, GSH) were measured in aliquots of perfusate sampled at different time intervals. Histological determinations and oxidative stress biomarkers in homogenized liver (AOPP; TBARS; nitric oxide derivatives, NO*_x_*; GSH and glutathione disulphide, GSSG) were also made in the tissue at the end. Results showed that both damage and oxidant indicators significantly decreased while antioxidant increased in hypothermic preconditioned livers. In addition, homogenized liver determinations and histological observations at the end of the protocol corroborate the results in the perfusate, confirming the utility of the perfusate as a non-invasive method. In conclusion, hypothermic preconditioning attenuates oxidative damage and appears to be a promising strategy to protect the liver against IR injury.

## 1. Introduction

During liver surgery and transplantation, a serious clinical problem arises: the transient ischemia and reperfusion injury (IRI) [1,2,3]. IRI is a complex damage attributable to various factors. First, when the donor liver undergoes the process of retrieval surgery followed by cooling storage, energy consumption is reduced. Effects associated with anaerobic metabolism included lactate acidosis [4], proteolysis [5] and sinusoidal endothelial damage [3] among others. During implantation, grafts are exposed to reperfusion, normothermia and oxygenation, a process that triggers the release of reactive oxygen species (ROS) and inflammatory mediators [3,6].

Due to the wide diversity of mechanisms involved in IRI, many therapeutic mechanisms have been suggested to prevent hepatic damage [7]. Pharmacological interventions [2,8,9,10] and the different preconditioning mechanisms are the most common strategies [11,12,13].

Recently, hypothermic oxygenated perfusion has been proven to be successful in protecting livers from reperfusion injury [14,15,16], in a mechanism due to a change in the mitochondrial redox state and subsequent decrease in ROS generation [15]. A therapeutic role for hypothermia has been demonstrated in many tissues and against diverse injuries [17] and protective effects were first suggested to be a consequence of cellular metabolism reduction, thus preserving ATP levels [18]. Reduction of oxidative stress as well as in antioxidant consumption has also been shown [19,20]. Hypothermic perfusion was also proven to work as a preconditioning agent in isolated hearts [21]: When hypothermic perfusion was applied prior to ischemia and reperfusion, improvement in the metabolic state of the tissue and decrease in oxidative stress damage was found [21]. The use of hypothermic perfusion (20 °C) in isolated livers resulted in protecting cellular integrity [22]. Moreover, our previous work demonstrated that hypothermic perfusion (22 °C) stimulated several mechanisms of cell protection, including antioxidants and the activation of lysosomal and proteasomal systems to repair oxidative damage [23]. These effects of hypothermic perfusion lead us to consider whether hypothermia could be used as a preconditioning model in isolated perfused rat liver (IPRL).

In the present study, we determined the effects of a short hypothermic perfusion (10 min at 22 °C) in isolated liver when applied before an ischemic/reperfusion episode by means of tissue damage indicators and oxidative stress parameters released in perfusate.

The main novelty of our work is the fact that hypothermia can be used as a preconditioning method. In addition, we have also shown that liver status can be monitored with non-invasive techniques, using aliquots of the effluent perfusate.

## 2. Results

**Figure 1:**
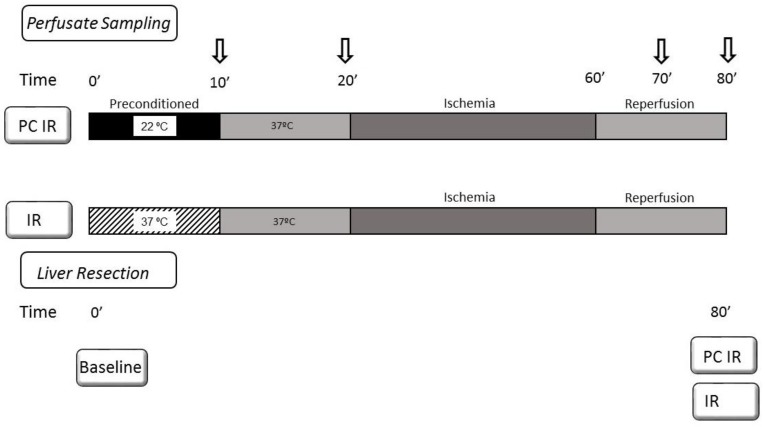
Schematic representation of experimental design. The IPR livers were randomly treated with hypothermic perfusion (22 °C) during 10 min (PC IR group) or perfused at 37 °C (IR group). Both groups were then submitted to 10 min at 37 °C. The perfusion was conducted under temperature-controlled conditions. The liver, kept moist in KHB solution, was placed in a temperature-controlled box, surrounded by a continuously stirred buffer at the set temperature (37 °C or 22 °C). Control of liver temperature was carried out with a thermocouple located at the catheter in the thoracic inferior vena cava. After the preconditioning period (20 min), ischemia (40 min) was induced by stopping the perfusion machine while keeping the liver-containing device surrounded by warm water to avoid cooling. Reperfusion was initiated by turning on the perfusion machine, circulating with warm buffer at 37 °C. Perfusate was sampled at different time intervals: after preconditioning (10 min), before ischemia (20 min), after ischemia/reperfusion (70 min) and at the end of reperfusion (80 min). At the end, liver extracts were also analysed to be compared to fresh resected livers.

We evaluated some parameters of tissue damage and oxidative stress in the perfusate collected in an isolated perfused rat liver (IPRL) model at different times during preconditioning, ischemia and reperfusion. The IPR livers were randomly treated with hypothermic perfusion (22 °C) for 10 min (hypothermia, PC IR group) or perfused at 37 °C (normothermia, IR group). Both groups were then submitted to 10 min at 37 °C followed by 40 min of ischemia and 20 min of reperfusion at 37 °C. Perfusate was sampled at different time intervals: after preconditioning (10 min), before ischemia (20 min), during reperfusion (70 min) and at the end of reperfusion (80 min). At the end, liver tissue and homogenates were also analysed and compared to fresh resected livers (see Figure 1).

### 2.1. Effect of Hypothermic Perfusion on Cellular Integrity

**Figure 2:**
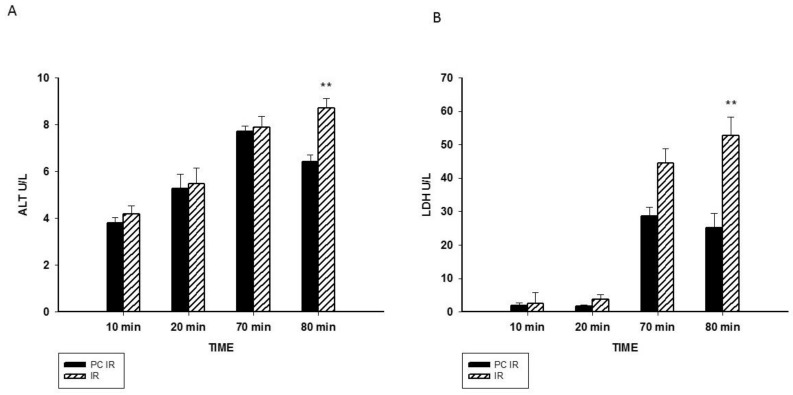
Effect of hypothermic perfusion on cellular integrity. (**A**) Alanine aminotransferase (ALT) in the perfusate; and (**B**) lactate dehydrogenase (LDH) in the perfusate. Data is mean ± SEM of six animals. Significantly different from corresponding PC IR values by Student’s *t* test: ** *p* < 0.01.

**Figure 3:**
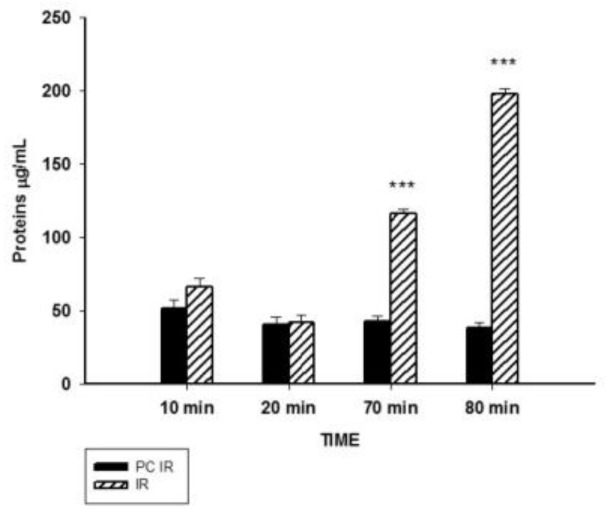
Effect of hypothermic perfusion on cellular integrity. Total protein content in the perfusate. Data is mean ± SEM of six animals. Significantly different from corresponding PC IR values by Student’s *t* test: *** *p* < 0.001.

As an index of cellular injury, alanine aminotransferase (ALT), lactate dehydrogenase (LDH) and the concentration of proteins in the perfusate were measured. ALT is mainly present in the cytoplasm of hepatocytes and its release increases with minor liver damage. LDH is a non-liver specific enzyme present in hepatocytes and non-parenchymal cells, used as a biomarker of general tissue-damage. Our results showed a significant increase of ALT and LDH after 20 min of perfusion (Figure 2A,B). The increase in released proteins was more significant, since it was observed earlier: at 70 min and 80 min of the experiment, during reperfusion (Figure 3). Thus, hypothermic preconditioning better preserves cellular integrity.

### 2.2. Effect of Hypothermic Perfusion on Biomarkers of Oxidative Stress in the Perfusate

**Figure 4:**
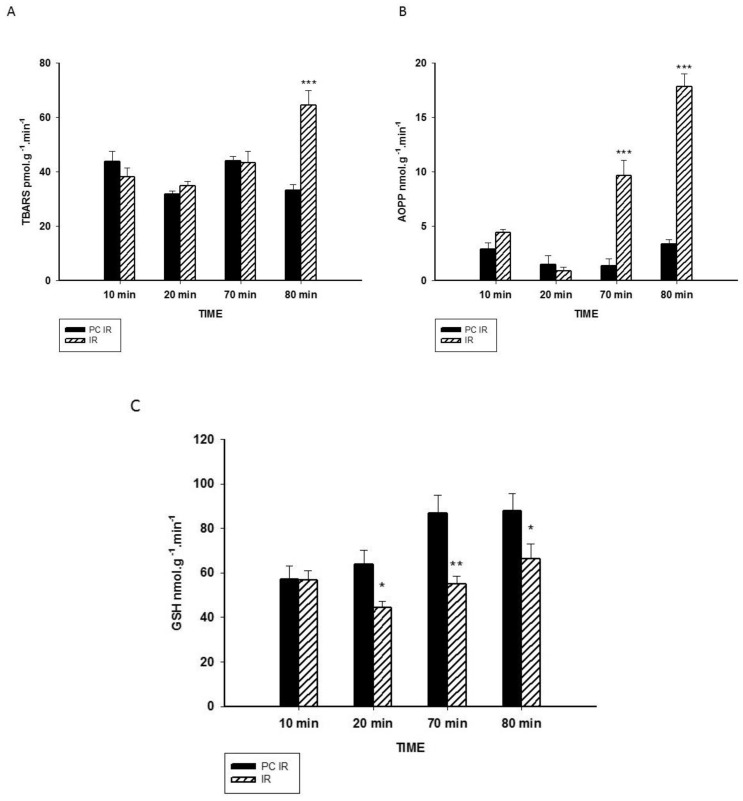
Effect of hypothermic perfusion on biomarkers of oxidative stress in the perfusate. (**A**) Lipid peroxidation (TBARS) (**B**) Advanced oxidation protein products (AOPP) and (**C**) GSH levels in the perfusate. Data is mean ± SEM of six animals. Significantly different from corresponding PC IR values by Student’s *t* test: * *p* < 0.05, ** *p* < 0.01 and *** *p* < 0.001.

As effects of oxidative stress, lipid peroxidation and protein oxidation were both measured. Lipid peroxidation was determined as the end product malondialdehyde by the thiobarbituric reactive substances (TBARS). In Figure 4A, we can see a significant increase in TBARS due to reperfusion in the IR group. We also measured the levels of advanced oxidation protein products (AOPP). AOPP has been identified as a biomarker of oxidative damage to proteins, detecting dityrosine and cross-linking protein products [24]. In Figure 4B, we can observe a dramatic increase in AOPP production due to the reperfusion in the IR group at both 70 and 80 min.

Under physiological conditions, oxidative stress can be avoided by the action of antioxidants. Glutathione (GSH) is one of the most ubiquitous antioxidant molecules [25,26] and, therefore, the levels of GSH in the perfusate were measured (Figure 4C). Reduced levels of GSH were found in the IR group at 20, 70 and 80 min of the experiment, indicating that the GSH decrease starts before ischemia and it remains stable in the reperfusion process.

### 2.3. Effect of Hypothermic Preconditioning on Oxidant/Antioxidant Parameters in Liver after Ex Vivo Warm Ischemia and Reperfusion Perfusion

**Figure 5:**
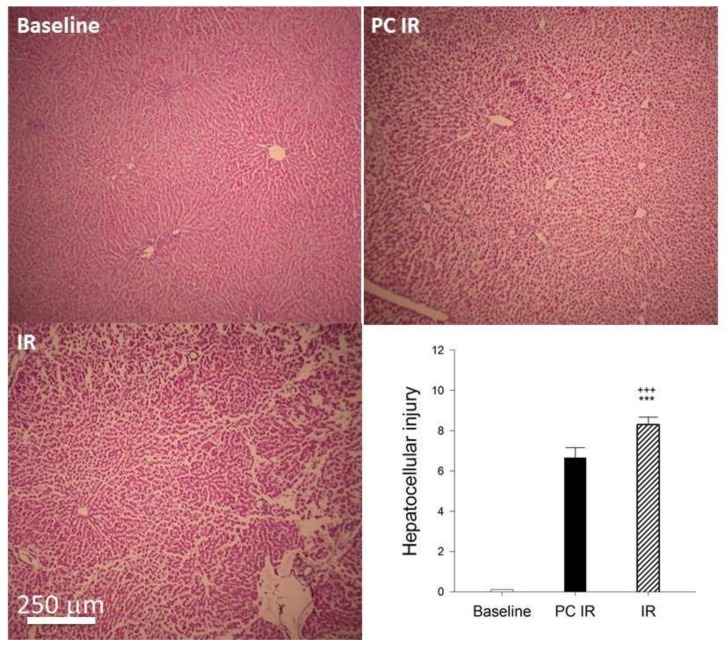
Histology hematoxylin-eosin staining. Photomicrographs at 10× magnification. Baseline: Fresh resected livers; PC IR: livers were preconditioned with hypothermic perfusion (22 °C) for 10 min and 10 min of normothermia previous to 40 min of ischemia and 20 min of reperfusion. IR: livers were perfused at normothermia, 37 °C for 20 min and then submitted to 40 min of ischemia and 20 min of reperfusion. Liver sections were stained with hematoxylin and eosin according to standard procedures. Scale bar, 250 µm. Tissue injury was scored as described in the Material and Methods section. Significantly different from corresponding Baseline values: ^+++^ *p* < 0.001. Significantly different from corresponding PC IR values: *** *p* < 0.001.

At the end of the ischemia/reperfusion protocol (80 min), livers were removed, and parameters were compared to fresh resected livers (Baseline); 5 μm sections of liver tissues fixed in formalin were stained with hematoxylin and eosin. Figure 5 shows the histology of the Baseline liver (fresh resected), PC IR and IR liver. In the images, we can see that the liver tissue in IR group has changed with large sinusoidal spaces between groups of cells, suggesting the loss of the extracellular matrix. In contrast, the tissue of the PC IR sample is better preserved.

Liver extracts were also homogenized, biochemical assays were performed and compared to fresh resected livers. Results in Table 1 showed that oxidant indicators measured as protein oxidation (AOPP), lipid peroxidation (TBARS) and nitric oxide (NO*_x_*) significantly increased in IR livers, when compared to fresh resected livers or to hypothermic preconditioned (PC IR) livers. It has been pointed out that nitric oxide, measured as nitrate plus nitrite, has a dual role: as a vasodilator regulates the hepatic microvascular perfusion, but it also acts as a pro-oxidant free radical. Total glutathione (that includes both forms, reduced and oxidized (GSH + GSSG), and the reduced form (GSH) decreased in IR livers, confirming glutathione depletion due to I/RI, that was avoided in the preconditioned livers.

## 3. Discussion

Hypothermia is defined as the situation in which the temperature of an organism is below its normal range (inferior to 37 °C). There are currently many studies on the therapeutic use of hypothermia in certain diseases. It is particularly recommended as a treatment in pathological or surgical situations [17,27]. In this context, hypothermia has improved the outcomes of patients with ischemic and traumatic brain accidents [28,29,30], as well as those who have had acute myocardial infarction [31,32] among others. Benefits of hypothermia may be related to a reduction in metabolism, affecting the catalytic enzymatic reactions and the oxygen consumed, thus maintaining ATP levels [18]. This is particularly important during an episode of ischemia and reperfusion since ischemia tends to the ATP exhaustion and reperfusion tends to the increase in the acid charge [33].

One of the feasible applications of hypothermia has to do with its role as a protective mechanism against the damage caused by ischemia/reperfusion (IR). Within the IR process, we can find an injury based on two fundamental components: (1) the lesion produced during the period of ischemia due to the lack of oxygen; and (2) the injury produced during the reperfusion process, mainly due to the synthesis of various inflammatory mediators and the generation of reactive oxygen and nitrogen species (RONS) after the restoration of the blood flow. Our previous work demonstrated that a brief hypothermic perfusion (22 °C) in isolated perfused rat liver (IPRL) induced cellular protection, related to antioxidant and proteasomal function [23].

These beneficial effects led us to explore the potential use of hypothermia as a preconditioning model for ischemia/reperfusion injury.

Our model of a short hypothermic perfusion (10 min at 22 °C) could fit well with the use of redox signalling as a physiological tool by the cell. In our previous assay, perfusion of livers with a cold buffer during 15 min resulted in a slight increase of both lipid peroxidation and protein oxidation in livers, evidencing a minor oxidative stress. At the same time, detoxifying systems such as glutathione or proteasomal systems appeared to be enhanced in livers [23]. Those adaptive intracellular mechanisms would be involved in the increased resistance of the hypothermia-preconditioned livers against IR challenge. For a cell, any adaptation strategy against a challenge will result either in death or survival, and mostly it is a matter of time. The results of this experiment are in accordance with this hypothesis. The use of a brief hypothermia perfusion as a “preconditioning” factor clearly triggered protective mechanisms in the cells that prepare the tissue for dealing with the ischemia and reperfusion process, therefore mitigating liver damage, decreasing tissue damage indicators (i.e., release of ALT, LDH and proteins into the perfusate), and cellular integrity preservation according to tissue histology. By looking at the levels of ALT and LDH in perfusate, it can be observed how there is a reduction in both enzymatic activities. The concentration of the enzymes in the cells is determined by the rate of their synthesis and degradation. In a previous work, we have shown that hypothermia causes the stimulation of the activity of cell proteolysis through the improvement of the proteasome system of ubiquitin and the lysosomal proteolytic machinery [23]. Therefore, we cannot exclude that an increase in the proteasomal degradation rate of many proteins in preconditioned livers with hypothermia would result in a lower enzyme activity of ALT and LDH. However, recently it has been reported that hypothermia-ischemia affects reaction velocity (*V*m) and also Michaelis constant (*K*m) in the enzymatic activity of LDH [34]. According to these significant data, hypothermia induces structural modifications of the LDH molecules, and not a decrease in their number. It has been suggested that the decrease in LDH activity maintains the level of low glycolytic lactate in tissues under ischemic conditions and hence contributes to the cellular protection of hypothermia [34].

As is well-accepted, oxidative burst induced by reperfusion impacts on biomolecules such as lipids and proteins. Moreover, ischemia and reperfusion decreased both total and reduced glutathione, the most important antioxidant molecule [35], in tissue. Because the increased recovery of GSH in the perfusate can indicate the toxicity resulting from the loss of cellular glutathione, the cellular contents in glutathione were analysed. As presented in Table 1, intracellular glutathione levels argue against depletion caused by greater elimination in pre-treated liver hypothermia, indicating protection against IR-induced depletion and suggesting that the increase in GSH levels in the perfusate could have another origin. In this sense, the lower degree of oxidative stress induced by short exposure to hypothermia (Table 1, Figure 4C) could promote a more GSH/GSSG balance in favour of GSH in the perfusate. Glutathione depletion impairs detoxification, increasing the formation of the advanced products of lipid peroxidation [36], coinciding with increasing release of AOPP and TBARS into the perfusate of IR group. In contrast, oxidative parameters decreased and antioxidants increased in the hypothermia PC IR group. Our results suggest that the protection due to hypothermia would be related to the preservation of the main antioxidant molecule glutathione [23,37].

Overall, this confirms our hypothesis that hypothermic preconditioning can be useful in avoiding oxidative damage; or in other words, the high levels of lipid peroxidation found in the liver during the ischemia reperfusion injury can be reversed thanks to the preconditioning methodology [38,39].

Moreover, the designed protocol allowed us to evaluate liver parameters in the eluted perfusate while contrasting them with the results in liver extracts after the IR protocol. We have found that the indices in the perfusate were corroborated by the tissue values. Therefore, our results can be applied in clinical terms, since perfusion values are faster to obtain and much less invasive than those that could be obtained from a tissue biopsy, so that it could be easily transferred to clinical transplants.

## 4. Materials and Methods

### 4.1. Animals and Liver Isolation and Perfusion

Adult male Sprague–Dawley rats (225–250 g body weight) were used in the study of isolated perfused rat liver (IPRL). Rats were fasted overnight and had free access to water. Rats were anesthetized with i.p. sodium pentobarbital (60 mg/Kg). Heart failure was induced by incision in the diaphragm and the liver was isolated and connected for perfusion in a non-recirculating system at a flow rate of 3 mL/min/g liver with Krebs-Henseleit buffer (KHB) (mM): 118 NaCl, 4.7 KCl, 1.2 MgSO_4_, 1.2 KH_2_PO_4_, 2.5 CaCl_2_, 25 NaHCO_3_, 20 Hepes (pH 7.4), aerated with 95% O_2_ and 5% CO_2_ The IPRL model was used as described by Vairetti [33] and others [40,41] with later modifications by our group [23]. The procedure was approved on the 8 February 2012 by the University of Barcelona Institutional Committee of Animal Care and Research (permission code DMAH 4125) and followed European Community guidelines.

Livers were randomly divided in 2 groups (*n* = 6). In the preconditioned group, livers were treated with hypothermic perfusion (22 °C) during 10 min (hypothermia preconditioning, PC IR group), and compared with livers perfused at 37 °C (Normothermia, IR group). The perfusion was conducted under temperature-controlled conditions. The liver, kept moist in KHB’s solution, was placed in a temperature-controlled box, surrounded by continuously stirred buffer at the set temperature (37 °C or 22 °C). Control of liver temperature was carried out with a thermocouple located at the catheter in the thoracic inferior vena cava. Both groups were then submitted to 10 min at 37 °C followed by 40 min of ischemia and 20 min of reperfusion at 37 °C. Ischemia was induced by stopping the perfusion machine while keeping the liver-containing device surrounded by warm water to avoid cooling. Reperfusion was initiated by turning on the perfusion machine, circulating with warm buffer.

Aliquots of 20 mL of perfusate were sampled at different times: 10 min (after hypothermia or normothermia perfusion); 20 min (before ischaemia); at 70 min (ischaemia plus 10 min of reperfusion); and at 80 min (ischaemia plus 20 min of reperfusion). All samples were immediately centrifuged at 800× *g* for 5 min at 4 °C to remove any cells. Fresh perfusate was used for lactate dehydrogenase (LDH) determinations and the remaining supernatant was frozen at −20 °C for lyophilisation. Samples of perfusate were lyophilized to dryness and diluted on the day of the assay according to the conditions of each test.

Freshly resected livers (Baseline) and livers from PC IR and IR groups were collected. From each liver some samples were kept fixed in formalin, and some samples were frozen in liquid nitrogen and stored at −80 °C until analysis for biochemical determinations.

### 4.2. Biochemical Assays in the Perfusate

As an index of tissue injury, alanine amino transferase (ALT) and lactate dehydrogenase (LDH) were measured using commercial kits (BioSystems, Barcelona, Spain) and expressed as U/L.

The total protein content in perfusate (μg/mL) was determined using the Bradford protein assay [42], and the formation of advanced oxidation protein products (AOPP) was spectrophotometrically measured at 340 nm following Witko–Sarsat’s method [24]. Results were obtained through a standard calibration curve using 100 μL of chloramine-T solution (0–100 μmol/L). AOPP levels were expressed as nmol·g^−1^·min^−1^ of Chloramine-T equivalents.

As a product of lipid peroxidation, we measured thiobarbituric acid reactive substances (TBARS), in accordance with the method of Yagi [43]. The chain-breaking antioxidant, butylated hydroxytoluene, and the iron chelator, EDTA, were used to prevent amplification of peroxidation during the assay. Results were expressed as TBARS in picomol·g^−1^·min^−1^ and compared to a standard obtained by the acid hydrolysis of tetraethoxypropane.

Glutathione groups in diluted lyophilized perfusate were measured using Ellman’s reagent in accordance with Hu [44] and were expressed as nmol·g^−1^·min^−1^.

### 4.3. Oxidant and Antioxidant Assays in Liver

Livers from PC IR and IR groups, and freshly resected livers (Baseline) were homogenized in 10% (*w*/*v*) with a teflon bar in a RIPA solution, (Tris 1 M, pH 7.5, Triton 10×, NaCl 5 M, NaF 1 M) containing antiprotease solution (aprotinin at 1.7 mg/mL, 2 µg/mL pepstatin, 2 µg/mL leupeptin and 1 mM phenylmethylsulfonyl fluoride and sodium ortovanadate at 1 mM). The suspension was centrifuged at 2000× *g* for 5 min and the cytosolic fraction discarded.

Advanced oxidation protein products (AOPP) in liver homogenates were assayed by a modification of Witko–Sarsat’s method [24,45]. The formation of AOPP was measured at 340 nm using 100 μL of Chloramine-T solution (0–100 μmol/L) as a standard calibration curve. AOPP was expressed as μmol/g wet weight.

Lipid peroxidation in livers was determined as the end-product malondialdehyde (MDA) by thiobarbituric reactive substances (TBARS) assay [46]. The formation of MDA-TBA adduct was fluorometrically measured at an excitation wavelength of 515 nm and an emission wavelength of 550 nm. The calibration curve was determined using tetraethoxypropane. Values are expressed in nmol/g wet weight.

Nitric oxide levels were measured as nitrate plus nitrite (NO*_x_*) in liver homogenates ultrafiltered by means of a 30 kDa molecular weight cut-off filter, using a colorimetric assay kit (Cayman Chemical Co., Ann Arbor, MI, USA). In the assay, nitrate was converted to nitrite using nitrate reductase and total nitrite was measured using the Griess reaction and expressed as μmol/g wet weight.

For measuring reduced glutathione (GSH) and oxidized glutathione (GSSG), liver was homogenized in a cold buffer containing 5 mm phosphate-EDTA buffer (pH 8.0) and 25% HPO_3_. The homogenates were ultra-centrifuged at 100,000× *g* and 4 °C for 30 min, and the resulting supernatant was used to determine GSH and GSSG concentrations, using the fluorescent probe o-phthalaldehyde. Fluorescence was determined at a wavelength emission of 420 nm and excitation at 350 nm [23,37]. Results are expressed as μmol/g wet weight.

### 4.4. Histology

Liver tissues were fixed in 10% neutral buffered formalin and embedded in Paraplast (Sigma Aldrich, Madrid, Spain), and 5 μm sections were stained with hematoxylin and eosin according to standard procedures. Samples were assessed according to the scoring system for nonalcoholic fatty liver disease proposed by Kleiner [47], with a minor modification: that is, considering the sum of steatosis (0–3), lobular inflammation (0–3), hepatocellular ballooning (0–2), together with dilatation of sinusoids (0–3).

### 4.5. Statistics

Results are expressed as means ± SEM of six animals. Data were processed using the statistical software GraphPad InStat (GraphPad Software, Inc., San Diego, CA, USA). The means of the experimental groups in perfusate samples were analyzed by the Student’s *t* test to identify significant differences between the two groups PC IR and IR at the same time intervals. Data of the liver biochemical and histological assays were analyzed by two-way ANOVA (when *p* < 0.05) using the post-hoc Student–Newman–Keuls test to identify significant differences between the Baseline, PC IR and IR groups.

## 5. Conclusions

In conclusion, hypothermic preconditioning at 22 °C in the liver is able to maintain the glutathione content and to attenuate the hepatic oxidative damage induced by ischemia/reperfusion and appears to be a promising strategy to precondition the liver against ischemic damage.

## Figures and Tables

**Table 1:** Effect of hypothermic preconditioning on oxidant/antioxidant parameters in liver after ex vivo warm ischemia reperfusion.

	Fresh Resected Baseline	Hypothermia PC IR	Normothermia IR
AOPP µmol/g wet weight	6.86 ± 0.19	4.33 ± 0.76 ^+^	6.36 ± 0.49 *
TBARS nmol/g wet weight	37.35 ± 1.32	37.22 ± 5.15	61.40 ± 9.45 *^+^
NO*_x_* µmol/g wet weight	0.16 ± 0.02	0.23 ± 0.06	0.43 ± 0.07 *^++^
GSH + GSSG µmol/g wet weight	1.66 ± 0.10	1.22 ± 0.25	0.79 ± 0.12 ^+^
GSH µmol/g wet weight	1.32 ± 0.07	1.11 ± 0.22	0.50 ± 0.07 **^++^

Livers were treated as followed: Fresh resected livers (Baseline); in hypothermia PC IR group, livers were preconditioned with hypothermic perfusion (22 °C) for 10 min and 10 min of normothermia (37 °C) previous to 40 min of ischemia and 20 min of reperfusion. IR livers were perfused at normothermia for 20 min and then submitted to 40 min of ischemia and 20 min of reperfusion. Oxidant indicators were the concentration of advanced oxidation protein products (AOPP), thiobarbituric acid-reactive substances (TBARS) and nitric oxide derivatives (NO*_x_*). Antioxidant status was evaluated as total (GSH + GSSG) and reduced glutathione (GSH). Data is mean ± SEM of six animals. Significantly different from corresponding Baseline values: ^+^ *p* < 0.05 and ^++^ *p* < 0.01. Significantly different from corresponding PC IR values: * *p* < 0.05 and ** *p* < 0.01.
